# Prospective Associations Between Leisure-Time Physical Activity and Cognitive Performance Among Older Adults Across an 11-Year Period

**DOI:** 10.2188/jea.JE20110084

**Published:** 2012-05-05

**Authors:** Po-Wen Ku, Clare Stevinson, Li-Jung Chen

**Affiliations:** 1 Graduate Institute of Sports and Health, National Changhua University of Education, Changhua City, Taiwan; 2 School of Sport, Exercise and Health Sciences, Loughborough University, Loughborough, United Kingdom; 3 Department of Exercise Health Science, National Taiwan University of Physical Education and Sport, Taichung, Taiwan

**Keywords:** exercise, physical activity change, latent growth model, cognition, dementia

## Abstract

**Background:**

Few studies have explored the relations between naturally occurring changes in physical activity and cognitive performance in later life. This study examined prospective associations between changes in physical activity and cognitive performance in a population-based sample of Taiwanese older adults during an 11-year period.

**Methods:**

Analyses were based on nationally representative data from the Taiwan Health and Living Status of the Elderly Survey collected in 1996, 1999, 2003, and 2007. Data from a fixed cohort of 1160 participants who were aged 67 years or older in 1996 and followed for 11 years were included. Cognitive performance (outcome) was assessed using 5 questions from the Short Portable Mental Status Questionnaire. Physical activity (exposure) was self-reported as number of sessions per week. The latent growth model was used to examine associations between changes in physical activity and cognitive performance after controlling for sociodemographic variables, lifestyle behaviors, and health status.

**Results:**

With multivariate adjustment, higher initial levels of physical activity were significantly associated with better initial cognitive performance (standardized coefficient β = 0.17). A higher level of physical activity at baseline (1996) was significantly related to slower decline in cognitive performance, as compared with a lower level of activity (β = 0.22). The association between changes in physical activity and changes in cognitive performance was stronger (β = 0.36) than the previous 2 associations. The effect remained after excluding participants with cognitive decline before baseline.

**Conclusions:**

Physical activity in later life is associated with slower age-related cognitive decline.

## INTRODUCTION

The rapid global trend of population aging is accompanied by the increasing prevalence of age-related disorders such as dementia.^1^ In East Asia, dementia incidence is projected to increase from 4.3 million new cases per year in 2005 to 19.7 million by 2050.^2^ Given the enormous impact of cognitive decline on individuals and their families,^3^ it is crucial to identify factors associated with such decline to help address this growing public health problem.

Accumulating evidence indicates that physical inactivity is a risk factor for age-related cognitive decline, and meta-analyses suggest that the risks of dementia^4^ and cognitive decline^5^ are reduced by late-life physical activity. The limitations of many early studies include poor control of underlying confounders such as baseline cognitive performance^6^ and reliance on a single baseline measure of physical activity. Given the variability of physical activity behavior over time, assessing exposure at additional time points provides more valid estimates of effect^7^ and allows the impact of changes in physical activity to be examined, which might be particularly important for older adults around the time of their retirement.^6^ Moreover, since cognitive function generally tends to decline with advancing age, the use of advanced analytical methods, such as the latent growth model (LGM), to assess change that is systematically related to the passage of time would provide a clearer understanding of the association between changes in physical activity and cognition.^8^ LGM is appealing not only because it can model intraindividual and interindividual change using a latent variable approach, but also because it permits exploration of the antecedents and consequences of change, which is not possible with traditional regression models.^9^

Studies of physical activity patterns in East Asia have consistently shown that, in contrast to similar populations in North America and Europe, leisure-time physical activity does not decrease with advancing age.^10^^,^^11^ However, there has been little research on the relationship between physical activity and cognitive decline in these populations.

The present study used LGM techniques to assess the relationship between changes in leisure-time physical activity and cognitive performance in an 11-year follow-up study of a nationally representative sample of Taiwanese older adults.

## METHODS

### Data and sample

This study was based on nationally representative data from the longitudinal Survey of Health and Living Status of the Elderly, which was undertaken by the Taiwan Department of Health using 3-stage equal probability sampling. Data were obtained from the Bureau of Health Promotion, Department of Health in Taiwan after approval by the Information Release Review Board. The 6-wave surveys began in 1989 (*n* = 4049 aged 60+) using household interviews, with follow-ups in 1993 (*n* = 3155 aged 64+), 1996 (*n* = 2669 aged 67+), 1999 (*n* = 2310 aged 70+), 2003 (*n* = 1743 aged 74+), and 2007 (*n* = 1268 aged 78+). The response rates of the surveys ranged between 88.9% and 91.8%.^12^ The 1996 survey was the first to include physical activity measures. From the original sample of 2669 participants interviewed in 1996, a fixed cohort of 1160 participants (50.5% male) aged 67 years or older at baseline with 11 years (1996–2007) of follow-up were included in the current analysis. The research design and sampling strategy have been reported in greater detail elsewhere.^13^^–^^15^

### Measures

Cognitive performance was assessed using the 10-item Short Portable Mental Status Questionnaire (SPMSQ).^16^ Because the number of items used in each survey varied (1996: 5 items, 1999: 8 items, 2003: 8 items, 2007: 9 items), only 5 items were used in this analysis, to ensure consistency. Respondents were required to recall their address, current age, the date, and day of the week, and to count backwards from 20 in steps of 3 a total of 4 times. A total score ranging from 0 to 5 was recorded based on the number of correct responses; higher scores indicated better cognitive functioning. The use of these questions as cognitive tests has been analyzed in other studies^14^^,^^17^ and has been validated for the Chinese version of the Mini-Mental State Examination (MMSE).^14^ The authors conducted a substudy to further examine the reliability and validity of this measure. The substudy included 96 community-dwelling older adults (mean age ± SD = 74.48 ± 6.45 years, male/female ratio = 30/66). Test-retest reliability with a 3-day interval was *r* = 0.69 (*P* < 0.001). Concurrent validity was examined by calculating the Spearman correlation between the 5-item scores and MMSE scores (Spearman ρ = 0.63, *P* < 0.001), and the results provided additional evidence of validity.

Regarding the assessment of physical activity, participants were asked “Did you usually engage in any kind of leisure-time physical activity?”. Four response categories were provided (none, 1–2, 3–5, and 6+ sessions per week), which were coded 0, 1.5, 4, and 7, respectively.^18^^,^^19^ The reliability and validity of this measure were assessed in the same sample described above. Test-retest reliability with a 3-day interval was *r* = 0.65 (*P* < 0.001). Concurrent validity was assessed by calculating the Spearman correlation between physical activity frequencies and triaxial accelerometer measures generated by the ActiGraph GT3X accelerometer (ActiGraph, Pensacola, FL, USA). Based on a 3-day activity record (2 weekdays and 1 weekend day), 1-week energy expenditure (or steps) was calculated as (2.5 × 2 weekdays) + (2 × 1 weekend day) (ρ = 0.36, *P* < 0.001; walking steps: ρ = 0.41, *P* < 0.001)^20^ and was comparable with findings from other studies of self-reported physical activity among older adults.^21^^,^^22^

Analysis of previous studies^6^^,^^23^^,^^24^ showed that potential confounders at baseline requiring adjustment were sociodemographic factors, ie, sex (1: female), age (67–99 years), education level attained (1: illiterate or primary school, 0: junior high school or above), cohabitation status (1: living alone, 0: not living alone), and self-perceived social support (1: unsatisfied vs. 0: average/satisfied); lifestyle behaviors, ie, alcohol drinking (1: current drinker, 0: never/former drinker) and smoking (1: current smoker, 0: never/used to smoke); and health status, ie, number of chronic diseases (0–6) and activities of daily living (ADL). ADL was assessed by examining 9 types of daily activities. Responses to each item were divided into 3 categories: 0 for no difficulty, 1 for some difficulty, and 2 for much difficulty. A composite index of difficulties in functioning was computed. Given that very few people had scores of 1 or higher (*n* = 121), only 2 categories were created (1: some/great difficulties; 0: no difficulty).

### Data analyses

#### Descriptive statistics

Means and standard deviations for frequency of physical activity and cognitive performance scores in 1996, 1999, 2003, and 2007 were estimated for the total sample and for different sex and age groups. One-way repeated measures ANOVA was used to compare scores across time, and 1-way ANOVA was used to assess group differences.

#### Latent growth model

**Figure 1.**
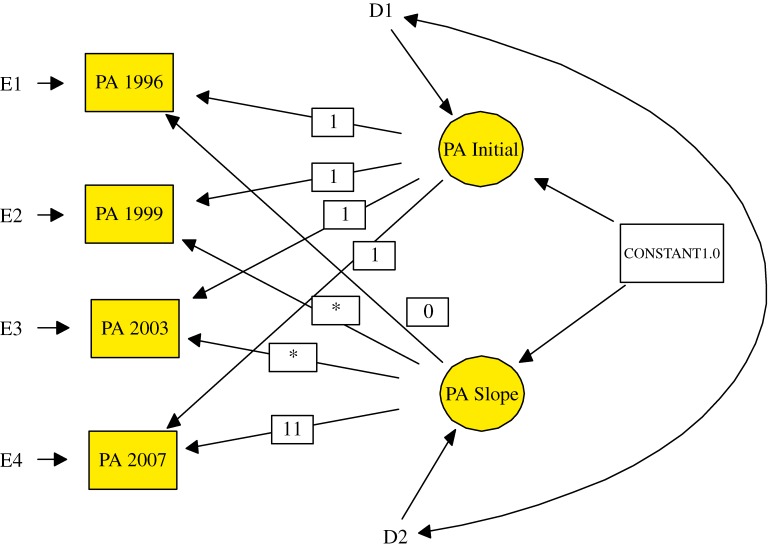
Specifications of the measurement model for estimating changes in physical activity. PA: physical activity. * denotes a free, as opposed to a fixed, parameter. E1–E4: Random error term representing intraindividual variance. D1–D2: Disturbance terms representing interindividual variance.

**Figure 2.**
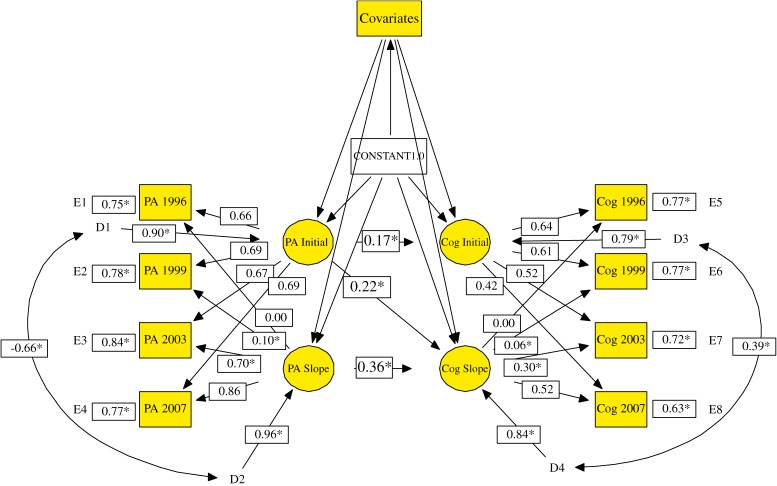
Standardized solution for testing the conditional latent growth model. * indicates statistical significance (Z-value > 1.96, *P* < 0.05). PA: physical activity; Cog: cognitive performance. In the interest of visual clarity, covariates are not shown. The covariates were sex, age, educational level, living status, social support, smoking, alcohol drinking, activities of daily living, and number of chronic diseases.

The LGM was adopted to examine the trajectories of changes in physical activity and cognition and to explore the relationships of physical activity with cognitive performance changes using the 4-wave data from 1996, 1999, 2003, and 2007.^25^^–^^27^ The specification of the LGM often consists of 2 steps.^27^ The first step (measurement model) is to analyze the trajectories of changes in physical activity and cognitive performance across time. It can be used to test for linear (0, 3, 7, 11), quadratic (polynomial growth) (0, 9, 49, 121), or unspecified curve (0, freely estimated, freely estimated, 11) (Figure 1) trajectories.^9^ The second step (structural model) is to assess the relationships of changes in physical activity with changes in cognitive performance. The present model was specified with physical activity as a time-varying predictor of change so as to control for participant’s concurrent status of physical activity and cognition in the 4 waves. Unconditional (without controlling for covariates) and conditional (controlling for covariates) models were examined sequentially (Figure 2). Because the inclusion of participants who had diminished cognitive performance before 1996 might affect estimates of physical activity–cognition relationships, sensitivity analysis was conducted. The model was rerun after excluding participants with cognitive decline from 1993 to 1996, which was defined as a difference between the 5-point scores from 1993 to 1996. Univariate normality was checked. None of the variables exceeded the criteria of extreme skewness (<3) or kurtosis (<8).^28^ The Mardia multivariate kurtosis coefficient was also checked (Mardia coefficients <p(p + 2), p: number of observed variables) and was satisfactory.^29^

For all models, the maximum likelihood missing data procedure was used and the Yuan-Bentler (Y-B) Scaled Test was adopted, which assumes that data are missing at random.^27^ Among the total sample (*n* = 1160), 312 participants had some missing data at the 4 time points. Two-sample *t*-tests and chi-square tests for independence were used to assess if missing data were related to physical activity, cognitive performance, and covariates at baseline. Cases with missing data tended to be inactive (3.55 sessions/week in cases with missing data vs 4.11 sessions/week in other cases; *t* = 2.55, *P* = 0.01), have lower cognitive scores (4.45 in cases with missing data vs 4.62 in other cases; *t* = 2.58, *P* = 0.01), be younger (cases with missing data: 73.31 years vs other cases: 74.41 years; *t* = 6.29, *P* < 0.001), and have difficulties in ADL (cases with missing data: 15.4% vs other cases: 8.6%; χ^2^(1) = 11.21, *P* = 0.002). These variables were all included in the subsequent conditional models.

The following indices were used to evaluate goodness of model fit when conducting LGM: chi-square test statistics, the comparative fit index (CFI) (>0.95), standardized root mean square residual (SRMR) (<0.05), the root mean square error of approximation (RMSEA) with 90% confidence interval (<0.08), and Akaike information criterion (AIC) (smaller values representing a better fit of a hypothesized model).^26^^–^^28^ The analyses were conducted using SPSS 17.0 (IBM, Chicago, IL, USA) and EQS 6.1 (Multivariate Software, Los Angeles, CA, USA).

## RESULTS

### Distributions of physical activity and cognitive performance

Physical activity patterns from 1996 to 2007 for the total sample and by sex and age group are shown in Table 1. The data show a curvilinear change in physical activity across time, with a slight increase in 1999 and a subsequent decrease. One-way repeated measures ANOVA suggested no significant change in physical activity across time (*P* = 0.10). Males were active more frequently (*P* < 0.05), and there were no age-related difference across the 4 time points (*P* > 0.05). Cognitive performance deteriorated across the period (*P* < 0.001). Males and younger age groups tended to have higher cognitive performance (*P* < 0.05).

**Table 1.** Levels of physical activity and cognitive performance in Taiwanese older adults, 1996–2007

Variables	1996			1999			2003			2007		
			
	*n*	Mean ± SD	*P*^a^	*n*	Mean ± SD	*P*^a^	*n*	Mean ± SD	*P*^a^	*n*	Mean ± SD	*P*^a^
**Physical activity**												
Total	1159	3.96 ± 3.28		1115	4.14 ± 3.18		1145	4.01 ± 3.22		1160	3.83 ± 3.18	
				Wilk’s lambda = 0.994, F (3, 1101) = 2.12, *P* = 0.10^b^, multivariate partial eta squared = 0.01								
Sex			<0.001			<0.001			0.003			0.046
Male	586	4.50 ± 3.17		560	4.62 ± 3.09		581	4.29 ± 3.20		586	4.01 ± 3.18	
Female	573	3.41 ± 3.31		555	3.65 ± 3.20		564	3.72 ± 3.22		574	3.64 ± 3.18	
Age			0.754			0.080			0.099			0.160
67–69	403	4.03 ± 3.28		390	4.43 ± 3.12		400	4.29 ± 3.19		404	4.05 ± 3.11	
70–74	493	3.88 ± 3.26		472	3.99 ± 3.20		485	3.88 ± 3.23		493	3.78 ± 3.21	
75+	263	4.01 ± 3.33		253	3.97 ± 3.22		260	3.82 ± 3.24		263	3.58 ± 3.22	

**Cognitive performance**												
Total	1079	4.58 ± 0.80		1050	4.51 ± 0.85		1037	4.24 ± 0.98		948	3.84 ± 1.20	
				Wilk’s lambda = 0.68, F (3, 847) = 134.20, *P* < 0.001^b^, multivariate partial eta squared = 0.32.								
Sex			<0.001			<0.001			<0.001			<0.001
Male	542	4.79 ± 0.56		530	4.77 ± 0.52		537	4.50 ± 0.80		496	4.07 ± 1.13	
Female	537	4.37 ± 0.94		520	4.24 ± 1.02		500	3.97 ± 1.07		452	3.58 ± 1.22	
Age			0.014			<0.001			<0.001			<0.001
67–69	384	4.67 ± 0.66		374	4.62 ± 0.71		378	4.44 ± 0.83		361	4.12 ± 1.08	
70–74	461	4.52 ± 0.88		441	4.51 ± 0.87		438	4.18 ± 1.01		395	3.77 ± 1.21	
75+	234	4.54 ± 0.84		235	4.32 ± 0.98		221	4.03 ± 1.07		192	3.46 ± 1.27	

^a^*P* value for 1-way ANOVA.

^b^1-way repeated measures ANOVA.

The number of cases does not always sum to 1160 due to missing data for some variables.

### Measurement model

Based on estimation using LGM, the trajectory of physical activity was best described by the unspecified curve model (ie, the parameters for assessing the trajectory were free rather than fixed) —assuming non-linear or non-quadratic change—and provided excellent fit indices (Table 2). The intercept of physical activity representing the initial status of physical activity (ie, in 1996) was 4.06 sessions per week. Consistent with the ANOVA results, the slope estimate (−0.02, *P* > 0.05) showed a nonsignificant decrease in the average rate of change in physical activity from 1996 to 2007.

**Table 2.** Standardized coefficients for measurement and structural models

Models	Parameters	Coefficients	z-value	Goodness of fit indices
**Measurement models**				
Trajectory of physical activity (*n* = 1160)	Intercept	4.06*	47.82	χ^2^(3) = 7.78, *P* = 0.05 χ^2^/df = 2.59; CFI = 1.00;
	Slope	−0.02*	−1.29	SRMR = 0.01; RMSEA = 0.04 (0.01–0.07)
Trajectory of cognitive performance (*n* = 1160)	Intercept	4.57*	194.04	χ^2^(3) = 9.56, *P* = 0.02 χ^2^/df = 3.19; CFI = 0.99;
	Slope	−0.07*	−24.23	SRMR = 0.01; RMSEA = 0.04 (0.01–0.08)

**Structural models**				
Unconditional model (*n* = 1160)	PA intercept → Cog intercept	0.37*	6.64	χ^2^(19) = 42.19, *P* = 0.002 χ^2^/df = 2.22; CFI = 0.97;
	PA intercept → Cog slope	0.27*	2.92	
	PA slope → Cog slope	0.36*	3.49	SRMR = 0.03; RMSEA = 0.03 (0.02–0.05)
Conditional model^a^ (*n* = 1160)	PA intercept → Cog intercept	0.17*	2.76	χ^2^(55) = 72.56, *P* = 0.06 χ^2^/df = 1.34; CFI = 0.98;
	PA intercept → Cog slope	0.22*	2.09	
	PA slope → Cog slope	0.36*	3.39	SRMR = 0.02; RMSEA = 0.02 (0.01–0.03)
Conditional model^a^ excluding participants with cognitive decline from 1993 to 1996 (*n* = 992)	PA intercept → Cog intercept	0.15*	2.00	χ^2^(57) = 68.18^b^, *P* = 0.11 χ^2^/df = 1.24; CFI = 0.99;
	PA intercept → Cog slope	0.22*	2.22	
	PA slope → Cog slope	0.29*	3.13	SRMR = 0.02; RMSEA = 0.01 (0.01–0.03)

*z-values greater than 1.96 indicate statistical significance (*P* < 0.05).

PA: physical activity; Cog: cognitive performance; CFI: comparative fit index; SRMR: standardized root mean square residual; RMSEA: root mean square error of approximation.

^a^The covariates were sex, age, educational level, cohabitation status, social support, smoking, alcohol drinking, activities of daily living, and number of chronic diseases.

^b^After excluding participants with cognitive decline from 1993 to 1996, change in cognitive performance was best described by the linear model. Two fixed parameters became free.

Although the trajectory of cognitive performance was well depicted by the quadratic model, the unspecified curve model yielded slightly better fit indices (AIC = 4.17 in unspecified model vs AIC = 4.63 in the quadratic model). The intercept of cognitive performance representing the initial status of cognitive function was 4.57. Accordingly, the negative slope estimate (−0.07, *P* < 0.05), indicates that there was a significant decrease in the average rate of change in cognitive performance during this period (Table 2).

### Structural model: unconditional modeling

The unconditional models (ie, without controlling for covariates) were specified to test the model in which initial status and slope factors for physical activity were associated with initial status and slope factors for cognitive function (Table 2; Figure 2). The unconditional model showed satisfactory goodness of model fit. First, the initial status of physical activity was significantly associated with the initial-status cognitive intercept in 1996 (path standardized coefficient β = 0.37), which suggested that participants with more frequent physical activity had, on average, higher cognitive scores than their less active counterparts at baseline. All analyses controlled for participants’ concurrent physical activity status in the 4 waves. Second, the path coefficient leading from the initial status of physical activity to the slope for cognitive status was significant (β = 0.27), which suggests that, as compared with less activity at baseline (in 1996), higher physical activity at baseline was related to a slower decrease in cognitive performance. Third, the rate of change in physical activity was significantly associated with the rate of change in cognitive performance (β = 0.36).

### Structural model: conditional modeling

The conditional model simultaneously controlled for sex, age, educational attainment, living status, social support, smoking, alcohol drinking, ADL, and number of chronic diseases, and demonstrated an excellent model fit (Table 2). As compared with the magnitude of associations between physical activity and cognitive performance in the unconditional model, the standardized coefficients were attenuated, but there were still significant positive associations between initial status factors (β = 0.17) and between slope factors (β = 0.36). Additionally, the path from the initial status of physical activity to the slope of cognition was significant (β = 0.22). Based on the conditional model, the additional model that further excluded participants with cognitive decline from 1993 to 1996 (*n* = 168) showed that although associations of physical activity and cognitive performance were slightly attenuated, they remained significant.

Regarding the residual variance associated with the intercept and slope for each latent factor (ie, “D”s), all parameters were significant (Figure 2). These findings indicate that there are strong interindividual differences in the initial scores for physical activity and cognition and in the changes of these scores. These strong interindividual differences indicate a need for further exploration of variability regarding the trajectories of change, especially with respect to the incorporation of more covariates into the model, to account for their variance. Additionally, the significant residual covariance (D1-D2, *r* = −0.66; D3-D4, *r* = 0.39) is evidence of interindividual differences in the association between initial status and slope in physical activity and cognitive performance.

The standardized path coefficients for the associations between the covariates and the initial status and slope in physical activity and cognitive performance are shown in Table 3. Individuals with lower initial levels of physical activity tended to be female (β = −0.20), illiterate or have primary schooling (β = −0.18), current smokers (β = −0.19), and to have some/great difficulties in ADL (β = −0.22). Sex, educational level, smoking, ADL, and chronic diseases were included as correlates of changes in physical activity. Females, older age groups, those with less education, those unsatisfied with social support, and those with some/great difficulties in ADL were more likely to have lower initial cognitive scores. Age, educational level, and living alone were identified as correlates of change in cognitive performance.

**Table 3.** Standardized coefficients for covariates in the conditional latent growth model

Covariates	Physical activity		Cognitive scores	
	
	Initial status	Slope	Initial status	Slope
Sex	−0.20*	0.12*	−0.28*	0.02
Age	−0.04	−0.02	−0.10*	−0.36*
Educational level	−0.18*	0.10*	−0.18*	−0.17*
Living alone	0.05	0.03	0.02	−0.15*
Social support	−0.06	0.06	−0.08*	0.03
Smoking	−0.19*	0.12*	0.04	−0.08
Drinking alcohol	0.04	−0.06	0.02	0.07
Activities of daily living	−0.22*	0.15*	−0.23*	0.05
No. of chronic diseases	0.02	−0.10*	0.04	−0.10

* indicates statistical significance (z > 1.96, *P* < 0.05).

The covariates were sex, age, educational level, living alone, social support, smoking, alcohol drinking, activities of daily living, and number of chronic diseases.

## DISCUSSION

The results over an 11-year period indicate that, in a large sample of older Taiwanese adults, initial physical activity and change in physical activity over time were associated with rate of decline in cognitive performance. These associations remained after adjusting for a comprehensive range of confounders, including sociodemographic variables, lifestyle behaviors, and health status. The effect persisted even after excluding participants with cognitive decline from 1993 to 1996. The implications are that involvement in physical activity in later life lowers the risk of future cognitive decline, and that reducing or increasing the frequency of physical activity is related to a respective concomitant decrement or improvement in cognitive performance.

The magnitudes of the associations observed here, although small to moderate,^30^ are consistent with those reported in previous reviews (ie, an approximately 20% to 40% lower risk for cognitive decline/impairment or dementia/Alzheimer disease).^4^^,^^5^^,^^31^ Because physical activity is a modifiable risk factor, there are important public health implications for evidence suggesting that even small benefits are achievable in terms of preventing cognitive decline through maintaining or increasing activity in later life.

Unlike the gradual decline in cognitive performance from 1996 to 2007, the magnitude of overall change in physical activity was small, ie, physical activity remained relatively stable across time in this population. Although we cannot rule out the possibility that the intervention effect from the first assessment of physical activity in 1996 influenced subsequent physical activity behaviors, our findings are compatible with previous evidence, which suggests that physical activity does not decrease substantially with advancing age in East Asia.^10^^,^^11^ This may be attributable to culture-specific physical activities, such as tai chi, which originate from the collectivistic traditions of East Asia and are common among older people, who often practice them daily.^10^^,^^32^

The pathways through which physical activity might influence cognitive functioning are not well understood. Potential neurobiological mechanisms include increased cerebral circulation and vascular health, neuronal plasticity, and neurotrophic factors.^33^ Moreover, physical activities during leisure time can provide enjoyment, fulfillment, and social interactions.^34^ The cognitive reserve theory posits that participation in cognitively stimulating leisure activities increases cognitive reserve, which might in turn maintain or improve cognitive functioning.^35^^,^^36^ However, although there has been accumulating evidence that physical activity is prospectively associated with lower risk of cognitive decline, it is also possible that cognitive deterioration inhibits future physical activity engagement. Future studies should explore the bidirectional associations between physical activity and cognition.

To our knowledge, this is the first observational study of physical activity and cognition to assess changes in both exposure and outcome variables over time. Additionally, it is the first study on this subject in a Taiwanese population. Key strengths of the study include the large and nationally representative sample, the retention of participants over an 11-year follow-up period, the range of potential confounders adjusted for in the analyses, and the use of LGM techniques. Moreover, the longitudinal design with 4 measures over 11 years made it possible to scrutinize non-linear relationships between physical activity and cognition, when many other studies simply assumed linear associations.

The attrition rate of the study sample was relatively high. The main reason for this was the relatively high mortality in this older population, given that the response rates across the 4 surveys were approximately 90%. The deceased participants tended to be male, older, less physically active, and smokers, and to consume less alcohol, live alone, and have lower cognitive performance and more difficulties in ADL and chronic diseases at baseline (data not shown). A limitation of the study is that information on physical activity was restricted to self-reported frequency and only represented leisure-time activity. Future studies should consider other components of physical activity such as duration, intensity, and type, and should investigate occupational, household, and transport activities. Additionally, the measure of cognitive performance was brief and did not assess all areas of cognitive functioning. Use of the complete versions of the SPMSQ and MMSE would be advisable in future research. Furthermore, the observational nature of the study prevents definitive conclusions about the direction of causality. Studies on the present subject are vulnerable to the possibility that low physical activity reflects existing subclinical neurodegenerative processes. Hence, it was important to control for baseline cognitive performance and its changes across time in this study.

In summary, the present results suggest that physical activity during later life is associated with slower subsequent age-related cognitive decline. This finding has implications for future physical activity and health promotion in older populations. Further well designed intervention studies are warranted to investigate the causal link between physical activity and cognitive decline.

## References

[1] Ferri CP, Prince M, Brayne C, Brodaty H, Fratiglioni L, Ganguli M, Global prevalence of dementia: a Delphi consensus study. Lancet. 2005;366:2112–7 10.1016/S0140-6736(05)67889-0 16360788 PMC2850264

[2] Access Economics. Dementia in the Asia Pacific region: the epidemic is here. Canberra: Access Economics; 2006.

[3] Tibaldi V, Aimonino N, Costamagna C, Obialero R, Ruatta C, Stasi M, Clinical outcomes in elderly demented patients and caregiver’s stress: A 2-year follow-up study. Arch Gerontol Geriatr. 2007;44:401–6 10.1016/j.archger.2007.01.056 17317482

[4] Hamer M, Chida Y Physical activity and risk of neurodegenerative disease: a systematic review of prospective evidence. Psychol Med. 2009;39:3–11 10.1017/S0033291708003681 18570697

[5] Sofi F, Valecchi D, Bacci D, Abbate R, Gensini GF, Casini A, Physical activity and risk of cognitive decline: a meta-analysis of prospective studies. J Intern Med. 2011;269:107–17 10.1111/j.1365-2796.2010.02281.x 20831630

[6] Coley N, Andrieu S, Gardette V, Gillette-Guyonnet S, Sanz C, Vellas B, Dementia prevention: methodological explanations for inconsistent results. Epidemiol Rev. 2008;30:35–66 10.1093/epirev/mxn010 18779228

[7] Martinson BC, Crain AL, Pronk NP, O’Connor PJ, Maciosek MV Changes in physical activity and short-term changes in health care charges: a prospective cohort study of older adults. Prev Med. 2003;37:319–26 10.1016/S0091-7435(03)00139-7 14507488

[8] Hox J, Stoel RD. Multilevel and SEM approaches to growth curve modeling. In: Everitt BS, Howell DC, editors. Encyclopedia of statistics in behavioral science. Chichester: Jogn Wiley & Sons; 2005. p. 1296–305.

[9] Preacher KJ, Wichman AL. Latent growth curve modeling. CA: Sage Publications; 2008.

[10] Ku PW, Fox KR, McKenna J, Peng T Prevalence of leisure-time physical activity in Taiwanese adults: results of four national surveys, 2000–2004. Prev Med. 2006;43:454–7 10.1016/j.ypmed.2006.04.011 16808968

[11] Bauman A, Ma G, Cuevas F, Omar Z, Waqanivalu T, Phongsavan P, ; Equity Non-communicable Disease Risk Factors Project Collaborative Group Cross-national comparisons of socioeconomic differences in the prevalence of leisure-time and occupational physical activity, and active commuting in six Asia-Pacific countries. J Epidemiol Community Health. 2011;65:35–43 10.1136/jech.2008.086710 20943821

[12] Taiwan Bureau of Health Promotion. Survey of health and living status of the middle aged and elderly in Taiwan survey report, 1989–2007. Taichung: Bureau of Health Promotion, Department of Health; 2010.

[13] Zimmer Z, Martin LG, Lin HS Determinants of old-age mortality in Taiwan. Soc Sci Med. 2005;60:457–70 10.1016/j.socscimed.2004.06.006 15550295

[14] Glei DA, Landau DA, Goldman N, Chuang YL, Rodriguez G, Weinstein M Participating in social activities helps preserve cognitive function: an analysis of a longitudinal, population-based study of the elderly. Int J Epidemiol. 2005;34:864–71 10.1093/ije/dyi049 15764689

[15] Ku PW, Fox KR, Chen LJ Physical activity and depressive symptoms in Taiwanese older adults: A seven-year follow-up study. Prev Med. 2009;48:250–5 10.1016/j.ypmed.2009.01.006 19297687

[16] Pfeiffer E A short portable mental status questionnaire for the assessment of organic brain deficit in elderly patients. J Am Geriatr Soc. 1975;23:433–41 1159263 10.1111/j.1532-5415.1975.tb00927.x

[17] Zimmer Z, Ofstedal MB, Chang MC Impact of cognitive status and decline on service and support utilization among older adults in Taiwan. Res Aging. 2001;23:267–303 10.1177/0164027501233001

[18] Kim Y, Kim H, Kim J, Lim Y Dietary intake based on physical activity level in Korean elementary school students. Nutr Res Pract. 2010;4:317–22 10.4162/nrp.2010.4.4.317 20827348 PMC2933450

[19] Birkeland MS, Torsheim T, Wold B A longitudinal study of the relationship between leisure-time physical activity and depressed mood among adolescents. Psychol Sport Exerc. 2009;10:25–34 10.1016/j.psychsport.2008.01.005

[20] Murphy SL Review of physical activity measurement using accelerometers in older adults: Considerations for research design and conduct. Prev Med. 2009;48:108–14 10.1016/j.ypmed.2008.12.001 19111780 PMC10071821

[21] Hagiwara A, Ito N, Sawai K, Kazuma K Validity and reliability of the Physical Activity Scale for the Elderly (PASE) in Japanese elderly people. Geriatr Gerontol Int. 2008;8:143–51 10.1111/j.1447-0594.2008.00463.x 18821997

[22] Harada ND, Chiu V, King AC, Stewart AL An evaluation of three self-report physical activity instruments for older adults. Med Sci Sports Exerc. 2001;33:962–70 10.1097/00005768-200106000-00016 11404662

[23] Lautenschlager NT, Almeida OP Physical activity and cognition in old age. Curr Opin Psychiatry. 2006;19:190–3 10.1097/01.yco.0000214347.38787.37 16612202

[24] Fratiglioni L, Paillard-Borg S, Winblad B An active and socially integrated lifestyle in late life might protect against dementia. Lancet Neurol. 2004;3:343–53 10.1016/S1474-4422(04)00767-7 15157849

[25] Duncan TE, Duncan SC An introduction to latent growth curve modeling. Behav Ther. 2004;35:333–63 10.1016/S0005-7894(04)80042-X

[26] Duncan TE, Duncan SC, Strycker LA. An introduction to latent variable growth curve modeling: Concepts, issues, and applications. 2nd ed. Mahwah, NJ: Lawrence Erlbaum Associates; 2006.

[27] Byrne BM. Structural equation modeling with EQS: Basic concepts, application, and programming. 2nd ed. Mahwah, NJ: Lawrence Erlbaum Associates; 2006.

[28] Kline R. Principles and practice of structural equation modeling. The Guilford Press; 2010.

[29] Bollen KA. Structural equations with latent variables. New York: Wiley; 1989.

[30] Cohen J. Statistical power analysis for the behavioral sciences. 2nd ed. Hillsdale, NJ: Lawrence Erlbaum; 1988.

[31] US Department of Health and Human Services. Physical Activity Guidelines Advisory Committee Report, 2008. Washington, DC: US Department of Health and Human Services; 2008.

[32] Hui SC, Morrow JR Level of participation and knowledge of physical activity in Hong Kong Chinese adults and their association with age. J Aging Phys Act. 2001;9:372–85

[33] Lista I, Sorrentino G Biological mechanisms of physical activity in preventing cognitive decline. Cell Mol Neurobiol. 2010;30:493–503 10.1007/s10571-009-9488-x 20041290 PMC11498799

[34] Teychenne M, Ball K, Salmon J Associations between physical activity and depressive symptoms in women. Int J Behav Nutr Phys Act. 2008;5:27 10.1186/1479-5868-5-27 18460191 PMC2397437

[35] Verghese J, Cuiling W, Katz MJ, Sanders A, Lipton RB Leisure activities and risk of vascular cognitive impairment in older adults. J Geriatr Psychiatry Neurol. 2009;22:110–8 10.1177/0891988709332938 19307322 PMC2692877

[36] Stern Y Cognitive reserve and Alzheimer disease. Alzheimer Dis Assoc Disord. 2006;20:112–7 10.1097/01.wad.0000213815.20177.19 16772747

